# Pedunculopontine Nucleus Area Oscillations during Stance, Stepping and Freezing in Parkinson’s Disease

**DOI:** 10.1371/journal.pone.0083919

**Published:** 2013-12-30

**Authors:** Valerie Fraix, Julien Bastin, Olivier David, Laurent Goetz, Murielle Ferraye, Alim-Louis Benabid, Stephan Chabardes, Pierre Pollak, Bettina Debû

**Affiliations:** 1 University Hospital, Department of Neurology, Grenoble, France; 2 Joseph Fourier University, Grenoble, France; 3 INSERM, U836, Grenoble Institute of Neuroscience, Grenoble, France; 4 Donders Institute for Brain, Cognition and Behaviour, Radboud University, Nijmegen, The Netherlands; 5 University Hospital, Department of Neurosurgery, Grenoble, France; 6 Clinatec- CEA-Leti, Grenoble, France; 7 Department of Neurology, Geneva University Hospital, Geneva, Switzerland; Oslo University Hospital, Norway

## Abstract

The pedunculopontine area (PPNa) including the pedunculopontine and cuneiform nuclei, belongs to the mesencephalic locomotor region. Little is known about the oscillatory mechanisms underlying the function of this region in postural and gait control. We examined the modulations of the oscillatory activity of the PPNa and cortex during stepping, a surrogate of gait, and stance in seven Parkinson’s disease patients who received bilateral PPNa implantation for disabling freezing of gait (FOG). In the days following the surgery, we recorded behavioural data together with the local field potentials of the PPNa during sitting, standing and stepping-in-place, under two dopaminergic medication conditions (OFF and ON levodopa). Our results showed that OFF levodopa, all subjects had FOG during step-in-place trials, while ON levodopa, stepping was effective (mean duration of FOG decreasing from 61.7±36.1% to 7.3±10.1% of trial duration). ON levodopa, there was an increase in PPNa alpha (5–12 Hz) oscillatory activity and a decrease in beta (13–35 Hz) and gamma (65–90 Hz) bands activity. PPNa activity was not modulated during quiet standing and sitting. Our results confirm the role of the PPNa in the regulation of gait and suggest that, in Parkinson disease, gait difficulties could be related to an imbalance between low and higher frequencies.

## Introduction

In advanced stages of Parkinson’s disease (PD), gait disorders such as freezing of gait (FOG) respond poorly to dopaminergic or surgical treatment. FOG is defined as a sudden inability to start or keep on walking, the patients describing their feet as glued to the ground [1]. The pedunculopontine nucleus area (PPNa) has been investigated as a new target for deep brain stimulation to treat gait disorders in PD. The PPNa includes the pedunculopontine and cuneiform nuclei and belongs to the mesencephalic locomotor region (MLR) [2], Animal studies suggested that it plays a major role in gait regulation and posture [5], [6]. In PD it has been reported that low frequency (between 10 and 70 Hz) chronic stimulation of this area could improve gait disorders and FOG in some, but not all, patients [7], [8], [9], [10], [11]. Functional imaging studies have shown that gait imagery and PPNa stimulation were associated with blood flow changes in the area of the MLR [12], [13], [14]. Furthermore, microrecordings performed during PPNa implantation have evidenced changes in neuronal activity during mimicked steps [15]. However, the precise role of the PPNa in gait control and FOG remains very elusive. Furthermore, it is not clear why in some patients FOG is relieved under PPNa stimulation, whereas others do not benefit from the procedure.

Regarding local field potentials (LFP), only one study recorded activity in the PPNa during gait in parkinsonian patients OFF levodopa [16]. These authors reported an increase in alpha (7–10 Hz) oscillatory activity related to gait speed, further supporting the idea that the PPNa plays a role in the regulation of gait in humans as in animals. However, only one patient had FOG episodes during the experiments, preventing group analysis of PPNa oscillations during FOG. Therefore, our first aim was to better describe the modulations of PPNa activity during FOG. We reasoned that if the PPNa plays a role in FOG, the oscillatory activity should be different between FOG episodes and fluent stepping. Because of technical limitations (cable length), we could not have the patients actually walk. We therefore used a step-in-place (SIP) task as a surrogate. SIP has the same basic features as gait: it involves rhythmic alternated stepping of the left and right legs, requiring bilateral coordination of the legs in the single and double phases of support, and shifting of the centre of mass over the stance leg at each step in order to keep balanced. It is a form of gait in which step length is reduced to 0. It has been shown to be very effective to induce FOG [17], [18] and to be strongly correlated with patients’ self-report of FOG [18].

In addition, no study so far has studied the involvement of the PPNa in stance control in humans. We therefore recorded the LFP activity of PPNa in PD patients at rest, during standing, and during SIP. We reasoned that if PPNa is involved in balance control in humans as in animals, we should see modulations in LFP oscillatory activity during standing versus sitting.

Finally, so far, the influence of levodopa on the PPNa oscillatory activity has been studied at rest and during self-paced upper limb movements [19], [20]. These authors reported that alpha activity was only present under levodopa at rest, and that levodopa increased the alpha peak preceding upper limb movement onset. The influence of levodopa on gait-related PPNa oscillatory activity has not been examined directly. In order to better understand the influence of levodopa on PPNa activity during standing and gait, or lack thereof, we tested patients both OFF and ON levodopa. Because patients undergoing PPNa electrode implantation suffer from levodopa-resistant FOG under chronic treatment condition, we expected to see little levodopa-induced modulation of PPNa oscillatory activities during SIP.

## Methods

### Patients

We included seven patients with PD and bilateral subthalamic nucleus (STN) stimulation. The clinical characteristics of the patients are shown in table 1. All patients underwent bilateral PPNa stimulation because of severe FOG in spite of otherwise efficient dopaminergic treatment and STN stimulation. Patient 6 had stopped taking levodopa for several years because of dyskinesia and he was only evaluated OFF levodopa. The study was approved by the Grenoble University Hospital Ethics Committee and written informed consent was obtained from each subject according to the Helsinki Declaration (trial: 06-CHUG-21). The effects of PPNa stimulation on FOG at 1-year in six of these patients have already been published [7].

**Table 1:** Patients’ characteristics.

Patient	Age at PDonset (years)	Age at STN surgery	Age at PPNasurgery	Disease duration (years)	LEDD(mg)	UPDRS III before PPNa surgery (/108)			
						OFF	MED	ON	MED
						OFF STN	ON STN	OFF STN	ON STN
1	55	64	68	13	1025	49	22	22	24
2	50	64	68	18	550	62	57	52	45
3	49	65	72	23	800	38	19	29	30
4	44	53	57	13	1170	32	16	18	12
5	31	53	59	28	400	40	19	22	18
6	27	47	56	29	–	46	18	ND	ND
7	36	52	61	13	800	63	32	23	10
**Mean**	**41.7**	**56.9**	**63**	**21.3**	**790.8**	**47.1**	**26.1**	**27.7**	**23.2**
**SD**	**10.5**	**7.3**	**6.3**	**6.7**	**286.2**	**11.8**	**14.6**	**12.4**	**13**

PD: Parkinson disease, STN: subthalamic nucleus, PPNa: pedunculopontine nucleus, LEDD: levodopa equivalent daily dose, MED: medication. ND: not done. Note that the mean LEDD is computed for the 6 patients under dopaminergic medication only.

### Electrode Implantation

**Figure 1:**
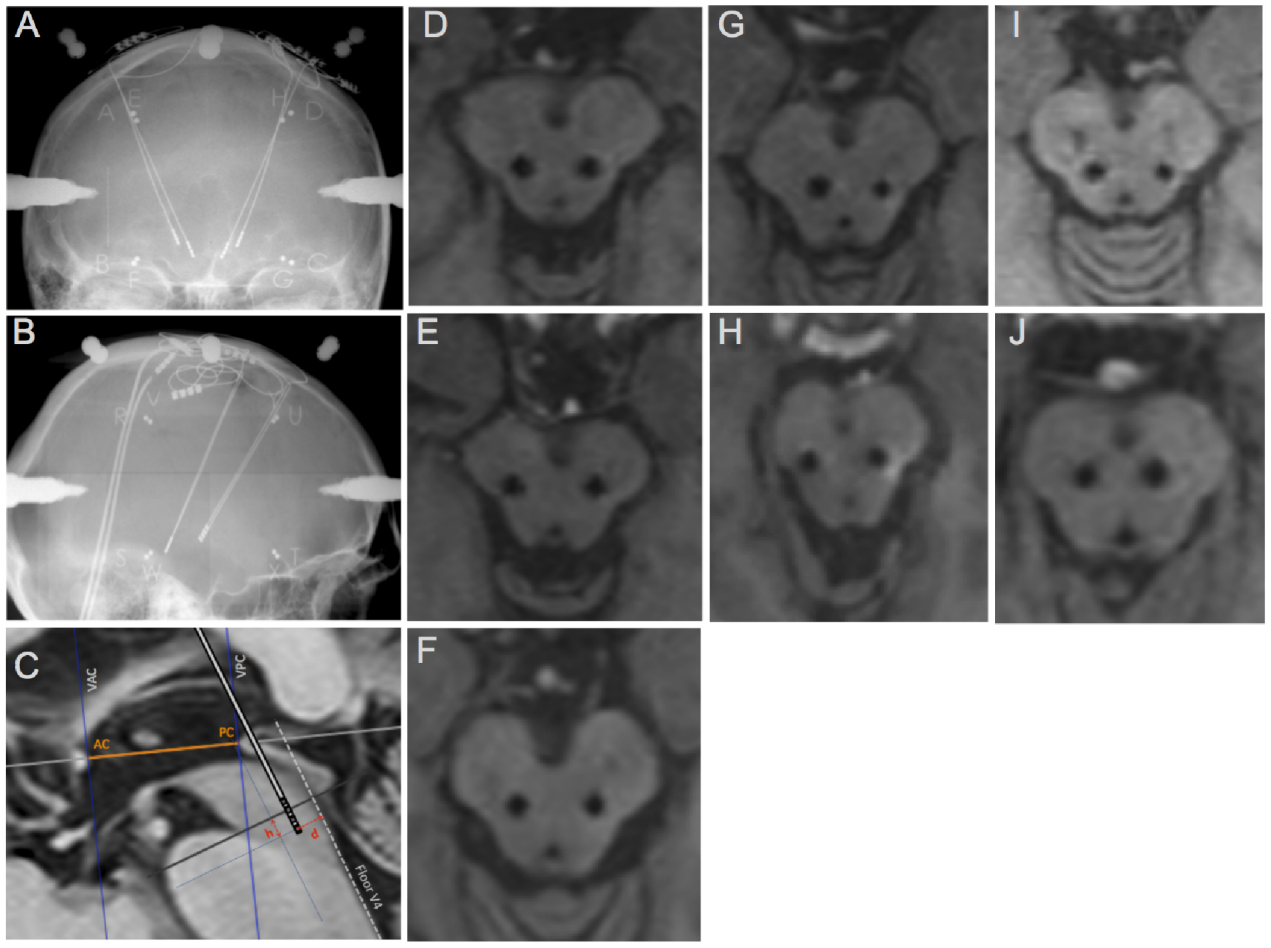
Localization of the DBS electrodes within the Pedunculopontine nucleus area (PPNa). A–B: Final intra-operative anteroposterior and lateral teleradiographies in patient 1 (D). The two medial and posterior electrodes are implanted in the PPNa while the two anterior and lateral leads are implanted in the subthalamic nucleus (STN). Note that the electrodes in PPNa are superimposed on the lateral view (see also Table 2). C: schematic representation of brainstem coordinates calculation on the mid-sagittal plane according to pontomesencephalic landmarks. D-J : PPNa DBS electrodes positions in the 7 patients on a 3D-T1-weighted magnetic resonance imaging reconstructed in the transverse plane of the brainstem at the pontomesencephalic junction level (h : distance to the pontomesencephalic line; AC : anterior commissure; PC: posterior commissure. d : orthogonal distance to the line prolonging the fourth ventricle line. V4: fourth ventricle).

The surgical procedure has been published elsewhere [7]. Briefly, the PPNa was targeted bilaterally using stereotactic T1 and T2 brain MRI and contrast ventriculography to define the bicommissural line and the fourth ventricle [15]. Intraoperative microrecordings and microstimulation were performed along two or three microelectrode trajectories. Quadripolar leads (DBS-3389, Medtronic, Minneapolis, MN, USA) were implanted along the trajectory with the greatest threshold for stimulation-induced side effects and in which cells were recorded. The final location of all contacts of the PPNa leads was checked using the coordinates of each contact on the final intraoperative teleradiography (Pixray, Bioscan system, Switzerland) plotted onto the preoperative stereotactic MRI using an image navigation software (Osirix, http://www.osirix-viewer.com) (Figure 1). The coordinates of the tip of the distal contact of the electrodes implanted in the PPNa are reported in table 2.

**Table 2:** PPNa electrodes coordinates.

			Pontomesencephalic line	
	Contact	Laterality X (mm)	Antero-posterior d (mm)	Depth (rostro-caudal) h (mm)
**Patient 1**	0	5.3	8.7	1.5
	4	6.0	8.7	1.5
**Patient 2**	0	6.7	9.5	0.8
	4	7.5	9.5	0.8
**Patient 3**	0	5.7	7.7	−1.3
	4	5.5	7.7	−1.3
**Patient 4**	0	2.6	6.5	−2.9
	4	5.0	6.1	−2.9
**Patient 5**	0	4.7	8.9	−1.7
	4	5.4	8.4	−1.7
**Patient 6**	0	4.5	9.8	−1.1
	4	4.9	9.8	−1.1
**Patient 7**	0	4.5	5.1	− 4.21
	4	7.0	5.1	− 4.21

The coordinates indicate the laterality of the tip of the distal contact of the right and left PPNa electrodes respectively; right and left distance (d) anterior to the line extending from the floor of the 4^th^ ventricle and the right and left distances to the pontomesencephalic (PM) line, defined as the line joining the pontomesencephalic junction and the caudal quadrigeminal plate end measured on the midline (−: below this line; +: above this line).

### Electrophysiological Recordings

We recorded the LFP of the PPNa from the quadripolar leads through extension cables four days after implantation and before connection to the neurostimulator. The bipolar PPNa LFP activity was recorded continuously from the adjacent four contacts (contacts 0–1; 1–2; 2–3) of each deep brain stimulation lead implanted in the PPNa. Cortical EEG activity was recorded from Fz and Cz using needle electrodes with an ear reference, using an audio–video–EEG monitoring system [SystemPlus™ software, Micromed, Teviso, Italy]. The choice of mesial electrodes was based on four arguments: 1) midline EEG is likely arising from the pre-supplementary motor area and the precentral gyrus, thought to be involved in locomotion [13], 2) gait and stance are symmetrical bilateral motor skills involving the lower limbs, which are represented along the mesial portion of the central sulcus, 3) FOG could be related to disruptive connectivity between midbrain locomotor regions and medial frontal regions [14], [21], 4) these electrodes were used by former authors to study cortico-subcortical coherence [22], [23], [24], [25]. All electrophysiological signals were sampled at 512 Hz ([0.1–200 Hz bandwidth]). All recordings were performed OFF STN stimulation.

### Experimental Procedure

Patients performed two to four 30-seconds trials under each of the following conditions: sitting at rest, standing and SIP. An examiner stood close to the patient during the standing and SIP recordings in order to prevent any fall. Assessments were first carried out after an overnight fasting and withdrawal of medication (OFF levodopa condition), and then repeated about 40 minutes after administration of 120% of the pre-surgery usual morning levodopa dose, so that patients were in their so-called best-ON motor state (ON levodopa condition).

### Behavioural Analyses

To record gait, inner soles (Stride Analyzer, B&L Engineering, Santa Ana, CA, USA) containing four footswitches (one each for the heel, big toe, first and fifth metatarsal heads), were placed in the patients’ shoes. The foot-floor contact data were collected using a telemetric acquisition system (Noraxon Telemyo 2400, Scottsdale, USA) with video recording synchronization. Insole’s recordings during SIP were obtained for five patients (because of technical problems, data for patient 2 were lost). Patient 3 could not perform the task because of major worsening of parkinsonian symptoms when STN stimulation was switched OFF.

**Figure 2:**
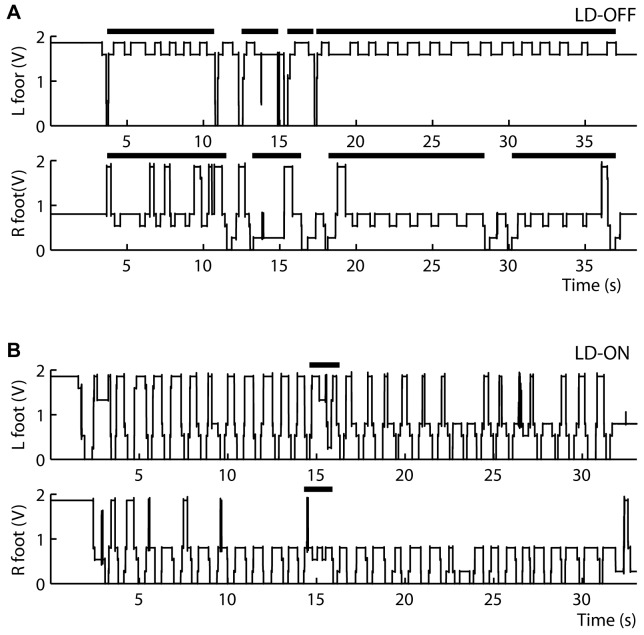
Foot switch recordings during a SIP trial OFF (upper panel) and ON (lower panel) levodopa (in patient 4). The voltage falls to zero when the foot is lifted off the floor. Horizontal black bars above the graphs indicate freezing of gait (FOG) episodes. The incomplete lifting of the foot when OFF DOPA reflects trembling FOG, i.e., the efforts of the patient to clear the foot from the floor when attempting to perform a step. ON DOPA, notice that for the left foot, the voltage does not reach its maximal value on most steps. This reflects the fact that the patient only loads on his forefoot when stepping.

Recordings from the insoles were processed off-line. The beginning and end of each FOG episode were marked on the foot contact data. Freezing of gait episodes were defined as an inability to perform effective stepping, i.e. completely lift the swing foot from the floor, for at least two seconds [26], [27]. This is easily seen on the insoles’ recordings, as the switch signal will not drop to 0 as long as the patient’s foot rests on the floor (see figure 2). The total duration of FOG for each trial was calculated as the sum of all freezing episodes, and expressed relative to the total duration of the trial (30 seconds), as percent. Our system did not enable synchronization of the insoles and electrophysiological recordings.

### Electrophysiological Analyses

Electrophysiological data were evaluated with custom-made functions developed under Matlab™ (Matlab R2007b, The Mathworks, Natick, MA, USA). For each of the six experimental conditions, bipolar derivations were computed between adjacent electrode contacts to suppress contributions from non-local assemblies and ensure that the bipolar LFP signals could be considered as originating from a volume in the vicinity of the two contacts [28].

We computed the power spectrum of LFP and EEG signals, as well as coherence between LFP and EEG, from all contact pairs using fast Fourier transform and Hanning windowing. To obtain a single value for cortex, we averaged together the power spectrum and coherence estimated at Fz and Cz. Spectral analysis was performed in the 1–90 Hz frequency range with a frequency resolution of 1 Hz, using a time window of 2 seconds duration that was moved every 0.2 s over the 30 s task periods. For each hemisphere (n = 14), we then selected the contact-pair that had the highest power in the alpha band (5–12 Hz), or in the beta band (13–35 Hz) if it was greater than in the alpha band [16]. We ascertained that there was no laterality effect and then computed relative power values in the alpha (5–12 Hz), beta (13–35 Hz) and gamma (65–90 Hz) frequency bands on the 14 electrodes. To do so, the average spectral power in each band was normalized by dividing each frequency band power value by the average power in the (5–90 Hz) frequency band. These relative power values were used for statistical analyses.

### Statistical Analyses

For each frequency band, we first examined the normality of distribution using Kolmogorov-Smirnov test. Homogeneity of variances was verified using a Bartlett test. As the assumptions for parametric statistics were met, we compared the mean relative power of the three different tasks (SIT, STAND and SIP) under the two medication conditions (OFF and ON levodopa) using an ANOVA with repeated measures. Post-hoc analyses were performed using the Newman-Keuls test. A p value ≤0.05 was considered significant. All statistical analyses were carried out using the Statistica™ software (Statistica, StatSoft, Tulsa, OK, USA).

## Results

### Behavioral Data

Under OFF levodopa, all subjects had FOG during SIP trials (figure 2). The mean duration of FOG episodes was 19±10.6 s (61.7±35.3% of trial duration). ON levodopa, only 2 of the 5 patients had freezing episodes during the tests, reducing the mean duration of freezing episodes to 3±3.2 s (7.3±10% of trial duration).

### PPNa Oscillatory Activity

Visual inspection of all power spectrum computed during the sitting trials revealed clear peaks in the alpha band (5–12 Hz) for most contact pairs (11 out of 14 contacts), and in the beta band (13–35 Hz) for the remaining three contact pairs.

**Figure 3:**
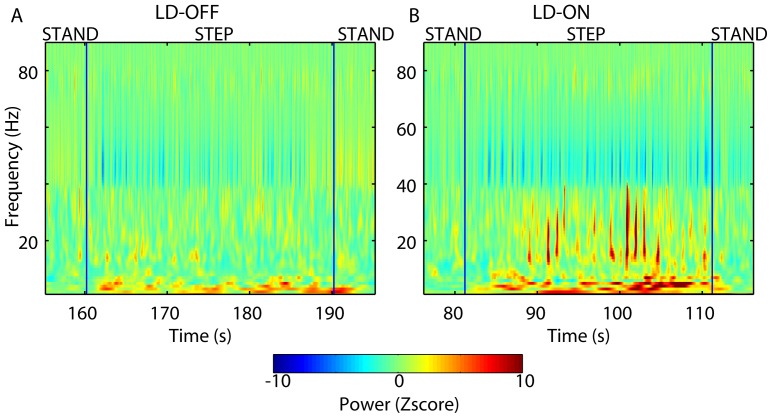
Pedunculopontine nucleus area (PPNa) oscillatory activity recordedduring a SIP trial, both OFF (A, LD-OFF) and ON levodopa (B, LD-ON). A–B (from contact pair 1–2 in patient 6): Time–frequency maps. The vertical blue lines mark the beginning and end of the SIP trial, preceded and followed by standing. Notice the increase in alpha and decrease in gamma spectral powers ON levodopa (B) during SIP *vs*. stand immediately preceding and following stepping (i.e., compared with before and after the vertical blue lines).

**Figure 4:**
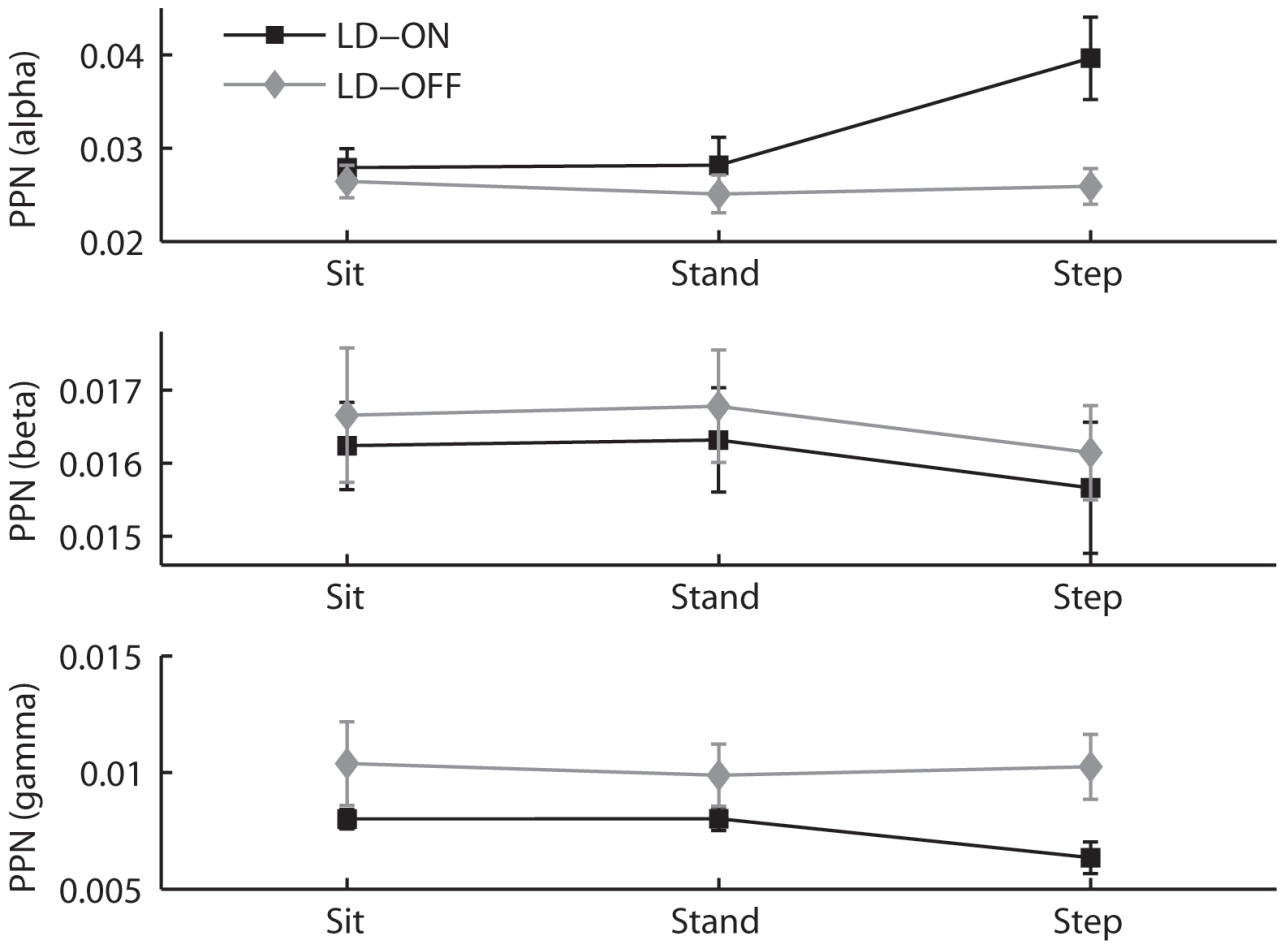
Average alpha, beta and gamma frequency powers in the PPNa during sitting, standing and SIP (n = 14 contact pairs). Error bars indicate standard errors. OFF levodopa, the overall duration of freezing of gait was 61% of the trials’ duration. Note the increase in alpha power when the patients manage to SIP while ON levodopa compared to OFF. There was a decrease in gamma power during SIP compared to standing and sitting ON levodopa.

Regarding the alpha band, the main effects of medication [F(1,9) = 20.24; p<0.01] and task [F(2,18) = 10.26; p<0.01] were significant, as was the interaction between the two factors [F(2,18) = 14.17; p<0.001]. Examination of the origin of this interaction showed that the alpha band power was significantly greater under the SIP ON levodopa condition than under any other condition (Newman-Keuls, p<0.01) (Figure 3 illustrates PPNa recordings obtained during a SIP trial). In other words, the alpha oscillatory activity was influenced by levodopa during the SIP task only (figure 4).

In the beta band, as can be seen on figure 4, analysis revealed a main effect of medication [F(1,9) = 13.74; p<0.01], beta power decreasing under levodopa, as well as a main effect of task [F(2,18) = 5.93; p<0.01]. Post-hoc analyses confirmed that, whatever the medication condition, the power was significantly reduced during the SIP task as compared to SIT and STAND (Newman-Keuls, p<0.05).

In the gamma band, the main effect of task and the interaction between task and medication were significant [Task: F(2,18) = 3.84; p<0.05; Interaction: F(2,18) = 18.97; p<0.001]. Examination of the origin of this interaction showed that the gamma band power was significantly reduced during SIP ON levodopa as compared to all other conditions (Newman-Keuls, p<0.01).

### Cortical EEG Oscillatory Activities

There was no effect of task, medication or interaction between task and medication in the alpha and beta bands (all p values>0.11). In the gamma band, whatever the medication condition, the power was significantly higher in the SIP condition than in the SIT condition [F(2,8) = 5.6; p = 0.03].

### PPNa-Cortex Coherence

**Figure 5:**
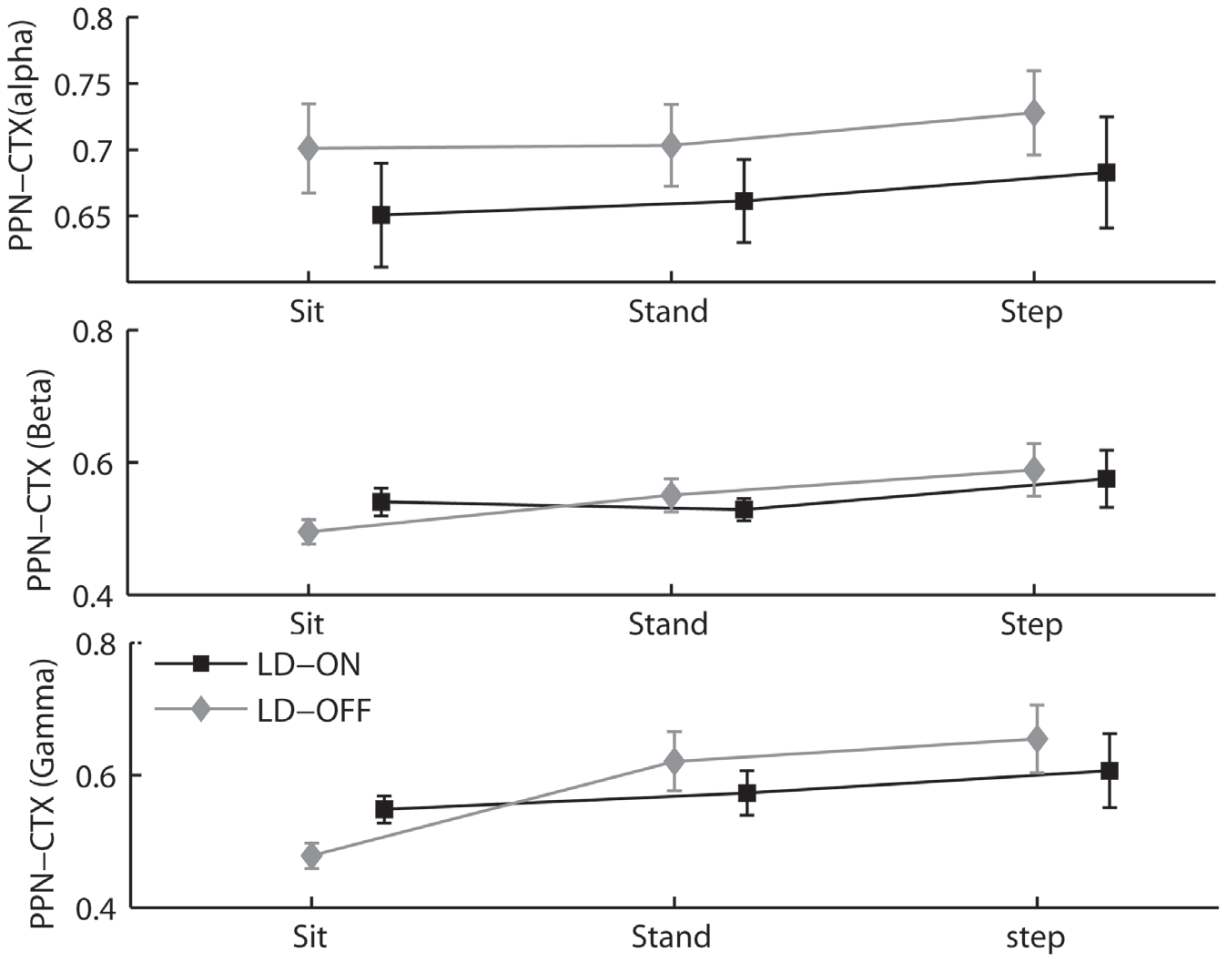
PPNa-cortex coherence. Average (n = 14 contact pairs) alpha (A), beta (B) and gamma (C) frequency coherence between the midline cortex and the PPNa during sitting, standing and SIP. Error bars indicate standard errors. Note a general trend to increasing coherence from sit to SIP in the alpha band and from sit to stand and SIP in the gamma band. This effect is mainly observed OFF levodopa.

We computed the average coherence between the PPNa contact pairs of each subject’s hemisphere and Fz-Cz (Figure 5). In the alpha band, there was a main effect of task on the PPNa-cortex coherence [F(2,18) = 6.02; p<0.05]. Post-hoc analysis revealed that, whatever the medication condition, the PPNa-cortex coherence increased during the SIP task compared to SIT and STAND (Newman-Keuls test p<0.05).

In the beta band, there was no effect of either medication, or task.

Finally, in the gamma band, the effect of task was significant [F(2,18) = 11.1; p<0.001]. Whatever the medication condition, the coherence between PPNa and cortex increased during the SIP and STAND tasks compared to SIT (Newman Keuls test; p<0.01).

## Discussion

We recorded the oscillatory activity of the PPNa and the cortex during sitting, standing and step-in-place in parkinsonian patients. The three main findings of our study are that: 1) alpha PPNa oscillations (5–12 Hz) increased ON levodopa only, during SIP, 2) PPNa gamma activity decreased from SIT to SIP, ON levodopa only, and 3) regarding the PPNa-cortical coherence, it increased in the alpha band during SIP and also increased in the gamma-band during STAND and SIP compared to SIT. Before discussing the significance of these results, we will examine the limitations of our study.

### Experimental Limitations

One limitation to this study is the number of patients included, which does not afford a strong power to the statistical analyses. However, PPN surgery currently remains experimental and our sample size is comparable to, or even greater than, that of other groups [16], [19], [20], [29].

Another concern relates to the task used to study PPNa activity during locomotion. Because of the limited length of the recording wires, it was not possible to record the activity during actual walking. However, we reasoned that SIP involved two of the major features of gait, namely balance control and rhythmic alternated activity of the two lower limbs. In fact, SIP resembles walking well enough to induce FOG very effectively [7], as confirmed by the behavioural results, with FOG occurring over 60% of the stepping-in-place sequences when patients were OFF levodopa. One could wonder why our patients were able to SIP ON levodopa during the tests, although they were operated on in the PPNa because of severe levodopa resistant FOG. Under usual daily life conditions, these patients actually suffered from FOG even ON levodopa. They were only able to SIP ON levodopa in this experiment, because the recordings were performed after administration of 120% of their usual levodopa equivalent morning dose, that is, while patients were under their so-called best-ON condition.

Unfortunately, our recording system did not enable synchronisation of the foot insoles and brain recording, and we could not compute correlations between FOG and oscillatory activity of the PPNa. In addition, we analysed PPNa and cortical activities while patients were either sitting or standing still in order to further distinguish the activity related to balance control from that related to stepping. Therefore the tasks used in this study enabled examining the involvement of the PPNa activity in the regulation of both balance and dynamical stepping.

Recordings were performed four days after surgery, and therefore it is possible that the recorded LFP activities might be altered by a microlesion effect, due to the short period between electrode implantation and recordings [30], [31]. Abosch et al. showed that electrode impedance and beta-band amplitude were lower in long term recordings from implanted DBS electrodes, but had a similar profile in response to movements as during acute recordings [32]. Thus, although it is considered that results from LFP analysis in the acute condition can be extended to the chronic condition [32], [33], [34], one should be cautious in extending the low frequency results of this study to the chronic stage.

Finally, one could wonder why our patients were able to SIP ON levodopa during the tests, although they were operated on in the PPNa because of severe levodopa-resistant FOG. Under usual daily life conditions, these patients actually suffered from FOG even ON levodopa. They were only able to SIP ON levodopa in this experiment because the recordings were performed after administration of 120% of their usual levodopa equivalent morning dose, that is, while patients were under their so-called best-ON condition.

### Pedunculopontine Nucleus Activity at Rest

#### Intrinsic local field potentials of the PPNa

Several authors have examined the electrophysiological characteristics of the PPNa at rest in patients with PD [16], [19], [20], [29], [35], [36]. Although there is no consensus on the electrophysiological signature for the LFPs of the PPNa, previous studies reported mainly alpha activity at rest with inconsistent beta activity [16], [19], [20], [29], [35], [36]. Our data recorded during sitting at rest are congruent with these reports, as we observed alpha peaks in 11 out of 14 electrodes and beta peaks in the remaining three. Aziz and Stein [37] suggested that beta activity in the PPNa could contribute to akinesia. In addition, a decrease, rather than an increase, in the beta power has also been described in the STN of PD patients, where this oscillatory activity is believed to have an antikinetic effect [38]. Thus, the presence of beta activity OFF levodopa and its decrease ON levodopa in the PPNa is congruent with the previous hypotheses regarding the involvement of this frequency band in parkinsonism.

#### The influence of levodopa on PPNa activity

Regarding the influence of levodopa, it has been reported to enhance the alpha and beta band oscillatory activities at rest in PD patients implanted in the PPNa for gait disorders [19], [20], [29]. These authors suggested that such modulation could have a physiological role. However, our own data failed to fully replicate this effect. While we observed an increase in alpha power activity under levodopa, especially when the patients are active, the beta power decreased ON levodopa as compared to OFF levodopa. Such discrepancies between Androulidakis’ [19], [20] and our results could stem from differences in the experimental conditions, patients’ characteristics and targeting.

Regarding patients’ characteristics, our patients had advanced PD but were successfully treated by STN stimulation except for severe FOG. The clinical and motor characteristics of these patients, including young-onset of disease, STN stimulation- and levodopa-responsive akineto-rigid syndrome with STN stimulation and levodopa resistant axial symptoms, is not typical of the natural evolution of PD but reflects the evolution of most PD patients with STN stimulation [39], [40], [41], [42]. They were offered PPNa surgery because under usual dopaminergic treatment, their FOG was not alleviated (contrary to what happens during a levodopa test based on a medication dose exceeding the usual patient’s dosage) and was their main complaint. It is not clear whether the patients of Androulidakis et al. had levodopa-responsive or levodopa-resistant FOG, which may be relevant to explain the differences in the results of the two studies. In addition, because they were successfully treated by STN stimulation, the usual levodopa daily dose (about 800 mg) of our patients was smaller than that reported for the patients included in Androulidakis’ studies (1000 to 1500 mg) [19], [20]. Thus, it is possible that the amount of levodopa used in the present study, although a largely supraliminal dose, was not sufficient to alter the PPNa oscillatory activity at rest. Yet, it was enough to alleviate FOG during the SIP task, confirming that the dosage was clinically relevant for these patients, as well as able to significantly decrease beta activity.

Another difference between our and Androulidakis’s patients is that ours were also stimulated in the STN, for an average of 6 years. Thus, one could envision that long term, plastic changes brought about by chronic STN stimulation might have a bearing on the present results. Yet, the literature on potential neuroprotective effects of DBS is very contradictory, and no report is available regarding the influence of STN stimulation on PPNa LFPs. In addition, while animal studies have documented bilateral connections between the STN and PPN, electrophysiological findings have yielded contradictory results. In humans, neuroanatomical, electrophysiological, and MRI probabilistic tractography studies have failed to demonstrate such direct links [21], [43], [44], [45]. Thus, hypothesizing that long term STN DBS did actually alter the LFPs of the PPNa remains highly speculative.

Differences in electrode location could also contribute to the divergent results across studies. Indeed, the actual PPN borders are not clearly delineated in humans [46], [47], [48] and the anatomy and atrophy of the mesencephalon may vary greatly from one brain to another [49]. The most suitable target to treat FOG in the MLR remains under debate and the localization of the electrodes within the PPNa differs from one patient to another and from one study to another [50], [51], [52]. Therefore we cannot exclude that difference in electrodes location within the MLR contributed to different outcomes across studies.

### Pedunculopontine Nucleus and Cortical Activities during Stand and Step-in-place Tasks

Regarding the influence of stepping movements, there was no modulation of the alpha PPNa power (5–12 Hz) when patients were mostly unable to perform the task OFF levodopa. Thevathasan et al. also failed to observe any change in the alpha power when PD patients walked OFF levodopa as compared to rest [16]. However, one important difference between the two studies is that the patients of Thevathasan et al. were able to walk OFF levodopa, while ours were not [16]. Our patients were only able to perform the task ON levodopa, and it was then associated with an increase in alpha PPNa power. These results are not as contradictory as they could seem at first sight. Indeed, Thevathasan et al. actually found a modulation of the alpha power when patients were walking, as they reported a correlation between alpha power and gait cadence on one hand, and an “attenuation in alpha power” in one patient who experienced FOG during the recordings on the other hand [16]. Thus, in the two sets of data, alpha power is increased in relation to effective gait and is comparatively reduced during FOG episodes. The main difference between the two studies could simply be the severity of the gait difficulties encountered by the patients.

At the behavioural level, the recorded modulation of the PPNa activity ON levodopa during SIP was associated with a greater success in performing the task, that is, a reduction of FOG episodes. This suggests that the increased alpha power is related to stepping rather than to freezing, in agreement with the results reported by Thevathasan et al. [16]. In addition, we did not observe any difference in alpha band activity when patients were standing vs. sitting. Altogether, the data suggests that the low frequency activity of the PPNa is related to the dynamical component of gait, i.e., rhythmical stepping. Hence, the restoration of gait ON levodopa in our patients could be related to the increase in alpha power, if the mechanisms underlying alpha band oscillatory activity are at least partly dopamine dependent. Conversely, OFF levodopa, failure of alpha oscillatory activity modulation could contribute to FOG.

In addition, we observed a decrease in beta band activity, especially during the SIP task. The reduction of these antikinetic oscillations under levodopa could contribute to the alleviation of FOG.

Our study is the first study in which gamma band oscillations were recorded within the PPNa. The PPNa gamma power decreased under levodopa only, during effective stepping-in-place compared to sit and stand. We did not observe any difference in gamma band activity when patients were standing vs. sitting, suggesting that the modulation of the high frequency activity of the PPNa could be related to rhythmical stepping as alpha band modulation.

The PPNa is not only involved in locomotion but is also part of the reticular ascending arousal system. We previously demonstrated that stimulating the PPNa could change alertness in some patients [53]. As stepping requires more attention than sitting and standing, and as levodopa may change alertness, it is possible that levodopa-induced PPNa alpha and gamma band modulations under levodopa are related to the increased alertness which may in turn contribute to locomotion.

Analyses revealed that the coherence between the activity of the PPNa and cortex in the alpha and gamma bands increased while patients stepped-in-place, as compared to sit. This effect was mainly observed when patients were OFF levodopa. Under this medication condition, as the PPNa alpha and gamma powers were respectively either unchanged or reduced by the task, the increased coherence appears to reflect mainly an increase in the phase-lock between the two structures. ON levodopa, the alpha power increased in the PPNa during the stepping task. It is thus not possible to know whether the increased coherence PPNa-cortex is related to the change in alpha power or in the phase locking between the PPNa and cortex oscillations. On the other hand, the gamma power decreased ON levodopa during the stepping task. This suggests that the increased coherence between the PPNa and the cortical gamma oscillations reflects an increase in the phase lock of these two structures. The hypothesis of phase-locking of PPN/cortex oscillatory activity is further supported by a recent study in MRI tractography showing a direct pathway between PPNa and frontal cortical regions [21]. As this pathway may be reduced in patients with FOG, it is likely that the estimated changes of coherence may be lower than in physiological conditions. This, together with our small sample size, may also explain why we did not observe any laterality effect, unlike what could be expected from the right-sided reduced connectivity reported by Fling et al. [21].

## Conclusion

In conclusion, we showed a reduced alpha band activity in the PPNa when patients were unable to step because of severe FOG. In contrast, under levodopa, that is, when patients were able to step in place, there was a selective increase in alpha band activity associated with a decrease in beta and gamma bands activity. We did not expect such effects as our patients had mainly levodopa-resistant FOG under chronic medication dosage. Yet, a higher than usual levodopa dose was able to both alleviate FOG and modify PPNa oscillatory activity. These results confirm the major role of the PPNa in the regulation of locomotion, consistent with the results of recent fMRI studies showing gait-related modulation of brain activity in the region of the PPN [14], [54]. They also suggest that an increase of low frequency oscillations and/or decrease in high frequency oscillations could be required to enable walking, and that, in PD, an imbalance between high and low frequencies could be responsible for the gait difficulties encountered by the patients. FOG episodes could be associated with a deficit in PPNa alpha power increase when patients attempt to walk. Levodopa could alleviate this symptom by restoring a more physiological modulation within the PPNa.

Finally, our results failed to reveal any specific modulation of the PPNa activity during quiet standing as compared to sitting. This reinforces the idea that the PPNa is mainly involved in gait control. This is consistent with ours and others’ clinical data showing an alleviation of FOG and falls related to FOG in some patients, without effect on posture or postural stability [7], [9].
